# Comparative analysis of composition and spatial variations in the foregut microbiota of male and female donkeys

**DOI:** 10.3389/fmicb.2025.1532265

**Published:** 2025-05-09

**Authors:** Yanwei Wang, Xiaotong Li, Zuowei Li, Qiaoqiao Han, Tong Hu, Qiyue Zhang, Honglei Qu, Haihua Zhang, Yangyan Qu, Donghui Shi, Qiugang Ma, Shimeng Huang

**Affiliations:** ^1^National Key Laboratory of Livestock and Poultry Nutrition and Feeding, College of Animal Science and Technology, China Agricultural University, Beijing, China; ^2^Laboratory of Feed grain Safety and Healthy Poultry Farming, Beijing Jingwa Agricultural Science and Technology Innovation Center, Beijing, China; ^3^College of Life Sciences, Shanxi University, Taiyuan, China; ^4^College of Animal Science and Veterinary Medicine, Jinzhou Medical University, Jinzhou, Liaoning, China; ^5^Hebei Key Laboratory of Specialty Animal Germplasm Resources Exploration and Innovation, College of Animal Science and Technology, Hebei Normal University of Science and Technology, Qinhuangdao, China; ^6^Beijing Sunlon Technology Bio-breeding Innovation Co. Ltd, Beijing, China

**Keywords:** gut microbiota, spatial variations, foregut, donkey, gender

## Abstract

Donkeys, as significant herbivorous mammals, also serve as valuable companion animals. Research on gut microbiota has underscored the essential role of microorganisms in maintaining gut health, supporting nutrient metabolism, and regulating immune function. As the gut microbiota is also shaped by factors such as sex, age, diet, environment and genetics, many studies have on the complexity and diversity of hindgut microbial communities, while few studies have focused on the foregut microbiota of donkeys. To address this gap, we conducted high-throughput sequencing of the highly variable V3-V4 region of the 16S rRNA gene from the donkey small intestine (duodenum, jejunum, and ileum) to characterize and compare microbiota composition and abundance between male and female donkeys. A total of 12 healthy and uniformly conditioned Dezhou donkeys (six males and six females, aged 2–3 years, 250 ± 10 kg in weight) were included in the study. The results showed that albumin (ALB), total cholesterol (TC), and high-density lipoprotein cholesterol (HDL-C) levels were significantly higher (*p* < 0.05) in the female group compared to the male group. Additionally, *α*-diversity indices (Ace, Chao, Simpson, and Sobs) were significantly different (*p* < 0.05) between the groups. The PCoA results indicated significant differences (*p* < 0.05) between male and female donkeys across all intestinal locations (R^2^ = 0.2372, *p* < 0.001). Similarly, the microbial composition of the jejunum (R^2^ = 0.1875, *p* = 0.019) and ileum (R^2^ = 0.1776, *p* = 0.007) showed significant differences between male and female donkeys. Additionally, Firmicutes, Fusobacteriota, Proteobacteria, and Actinobacteriota were the dominant phyla across all gut regions. In male and female donkeys, key genera included *Lactobacillus*, *Streptococcus*, *Sarcina*, and *Escherichia-Shigella*. Linear discriminant analysis effect size (LEfSe) analysis revealed gender-specific enrichment, with *Clostridium_sensu_stricto_1*, *Acinetobacter*, and *NK4A214_group* dominant in female duodenum and jejunum, while *Streptococcus* and *Erysipelotrichaceae_UCG-002* were enriched in males. Similarly, female ileum had enriched *Amnipila*, *Terrisporobacter*, and *Luteimonas*, whereas males showed higher levels of *Sarcina* and *Streptococcus*. *Blautia* and *Mogibacterium* were enriched in female duodenum and jejunum, while *Fusobacterium*, *Actinobacillus*, and *Moraxella* were more abundant in male ileum. These findings characterize the gut microbiota of healthy donkeys and provide novel insights into the differences between male and female donkeys, offering previously unknown information about donkey gut microbiota.

## 1 Introduction

Many countries around the world have a long history of donkey farming, especially China, where the number of donkeys raised is substantial (Seyiti and Kelimu, 2021). Donkeys contribute significantly to livelihoods and agriculture (Ravichandran et al., 2023). The Dezhou donkey, one of the top five excellent donkey breeds in China, is primarily found in Dezhou City and the surrounding areas of Shandong Province. In 2011, the Dezhou donkey was included in the national list of livestock and poultry genetic resources for protection, and it is recognized as an excellent local breed in Shandong Province, China (Wang et al., 2023). The Dezhou donkey is a medium-sized, well-proportioned breed with strong limbs, highly valued for both its service and meat production (Wang et al., 2023). It is known for its tolerance to roughage, strong adaptability, and good disease resistance. Donkeys play a significant role in various sectors, and their meat is known for its delicious, delicate flavor, being rich in protein, amino acids, and other nutrients (Seyiti and Kelimu, 2021; Wang et al., 2023). In addition, the composition of donkey milk is more similar to that of human milk, including lactose, lipids and proteins, etc. Due to its chemical and nutritional characteristics, it has some potential value in the medical and food fields (Bertino et al., 2022). In recent years, donkey farming in China has attracted increasing attention, with extensive research conducted on donkey growth, reproduction, gut health, and meat quality (Man et al., 2023), highlighting the need for further studies on their nutritional systems to promote sustainable development of the industry.

The intestine plays a key role in digestion and nutrient absorption, with its structure and function closely linked to animal growth and health (Ma et al., 2022; Zhang et al., 2024). This is particularly important for herbivores, which rely on gut bacteria to ferment plant fibers into volatile fatty acids, their main energy source (Husso et al., 2020; Dalile et al., 2019). Research shows that gut microbes significantly influence biometabolic phenotypes, impacting nutrient absorption, tissue development, and immune function (DuPont and DuPont, 2011; González Olmo et al., 2021). Gut microbes are also associated with various diseases; for instance, in horses with colitis, Bacteroidetes were predominant (40%), while healthy horses were dominated by Firmicutes (68%) (Costa et al., 2012). Beyond digestion, the gut microbiota is increasingly recognized for its role in nutrient utilization, digestive tract development, and immunity (Schluter et al., 2020). A recent study showed that donkeys from different regions had different functional differences due to differences in gut microflora. For example, Dezhou donkeys exhibited strong glucose conversion ability and Shigatse donkeys exhibited strong glucose metabolism and utilization ability, which makes them better adapt to the environment (Guo et al., 2023; Liu et al., 2020). Additionally, gender differences have been identified as important factors that interfere with the gut microbiota, and this has been confirmed in humans and mice (Ding and Schloss, 2014; Elderman et al., 2018). As the gut microbiota is shaped by factors such as sex, age, diet, environment and genetics, many studies have shown that fermentation of the hindgut makes its microbial community more complex and diverse, and few studies have focused on the donkey gut microbiota, particularly the foregut. More studies are needed to explore the gut and fecal microbiota of healthy donkeys, further research is necessary to reveal the microbial colonization patterns in the foregut of male and female Dezhou donkeys.

In this study, the foregut of 12 adult healthy Dezhou donkeys, including six male and six female donkeys, was examined to further explore the characteristics and functional prediction of the contents of the duodenum, jejunum and ileum, and to provide a scientific basis for further revealing the influence of sex on the digestive mechanism of the donkey gastrointestinal tract.

## 2 Materials and methods

### 2.1 Animal ethics statement

In this study, the utilization of animals in this study adhered to rigorous ethical standards and was formally approved by the Animal Care and Use Committee of China Agricultural University (Approval no.: AW81704202-1-1).

### 2.2 Experimental design and data collection

A total of 12 healthy and uniformly conditioned Dezhou donkeys (age: 2–3 years; body weight: 250 ± 10 kg) were selected for this study, comprising six males and six females. All donkeys were raised under the same breeding conditions at Shandong Dong’e Ejiao Co., Ltd., with a standard farm-provided ration offered ad libitum, along with free access to water. The donkeys were housed in single semi-open pens and fed twice daily at 07:00 and 19:00. During this period, none of the donkeys received probiotics or antibiotics for at least 3 months.

All Blood samples were collected in tubes (5 mL) from a jugular vein before feeding. Serum samples were obtained after centrifugation at 3,000 × g for 10 min at 4°C and then snap frozen in liquid nitrogen, awaiting subsequent analysis. The donkeys were slaughtered following a 12-h fast, after which the carcasses were carefully dissected and the intestinal organs removed. Samples were promptly collected from different intestinal locations (duodenum, jejunum, and ileum) of each donkey. All contents were collected, handled, and stored aseptically to prevent contamination. Samples were placed in 5 mL centrifuge tubes, immediately preserved in liquid nitrogen, and then transported to −80°C storage for long-term preservation until DNA extraction.

### 2.3 Serum biochemical indices

The levels of alanine aminotransferase (ALT), aspartate aminotransferase (AST), alkaline phosphatase (ALP), total protein (TP), albumin (ALB), total cholesterol (TC), total triglyceride (TG), high-density lipoprotein cholesterol (HDL-C), and low-density lipoprotein cholesterol (LDL-C) were measured using a chemistry analyzer (Commercial Kit, Nanjing Jiancheng Bioengineering Institute, China) following the manufacturer’s recommended procedures.

### 2.4 DNA sequencing and processing

Microbial genomic DNA was extracted from 12 samples using the E.Z.N.A.® Soil DNA Kit (Omega Bio-tek, Norcross, GA, USA). The extracted DNA served as a template to amplify the V3-V4 hypervariable region of the bacterial 16S rRNA gene, using universal primers 338F (5′-ACTCCTACGGGAGGCAGCA-3′) and 806R (5′-GGACTACHVGGGTWTCTAAT-3′). The amplified products were detected by agarose gel electrophoresis (2% agarose), recovered using the AxyPrep DNA Gel Recovery Kit (Axygen Biosciences, Union City, CA, USA), and quantified with a Qubit 2.0 Fluorometer (Thermo Fisher Scientific, Waltham, MA, USA) to pool in equimolar amounts. Amplicon libraries were sequenced on the Illumina HiSeq 2,500 platform (Illumina, San Diego, CA, USA) for paired-end reads of 250 bp. The amplicons were purified from agarose gels using the AxyPrep DNA Gel Extraction Kit (Corning, Glendale, USA), pooled in equimolar amounts, and sequenced on an Illumina MiSeq platform (Illumina, San Diego, USA) following standard protocols by Majorbio Bio-Pharm Technology Co., Ltd. (Shanghai, China).

Illumina sequencing data were filtered, denoised, concatenated, and de-embedded using QIIME2 to obtain high-quality sequencing data, with each sample region yielding ≥50,000 effective sequences for subsequent bioinformatics analysis. Tags were clustered into ASVs using DADA2. ASV taxonomic assignments were conducted by the RDP classifier (version 2.2) and annotated in the Silva1 database. Alpha diversity indices, including ACE, Chao, Shannon, and Sobs, were calculated using QIIME2 and the R package vegan (v2.5.6). A Venn diagram showing the number of shared and unique ASVs among the different intestinal segments was constructed using the VennDiagram package in R (v3.1.1). Principal Coordinate Analysis (PCoA) and permutational multivariate analysis of variance (Adonis) were carried out using R software (version 3.2.1).^1^ Genus-level microbial differences between different intestinal segments were analyzed using vegan v3.5.1, with comparisons made using the Wilcoxon rank sum test or Kruskal-Wallis rank sum test and pairwise comparisons to identify specific variations.

### 2.5 Statistical analysis

All statistical analyses were conducted using GraphPad Prism (version 9.0; GraphPad Software, La Jolla, CA, USA). Differences were evaluated by groupwise comparisons using Student’s *t*-test. Data are shown as means ± standard errors of the mean (SEMs). *p* < 0.05 were considered statistically significant.

## 3 Results

### 3.1 Differences in serum protein metabolism and lipid metabolism indices between adult female and male donkeys

**Figure 1:**
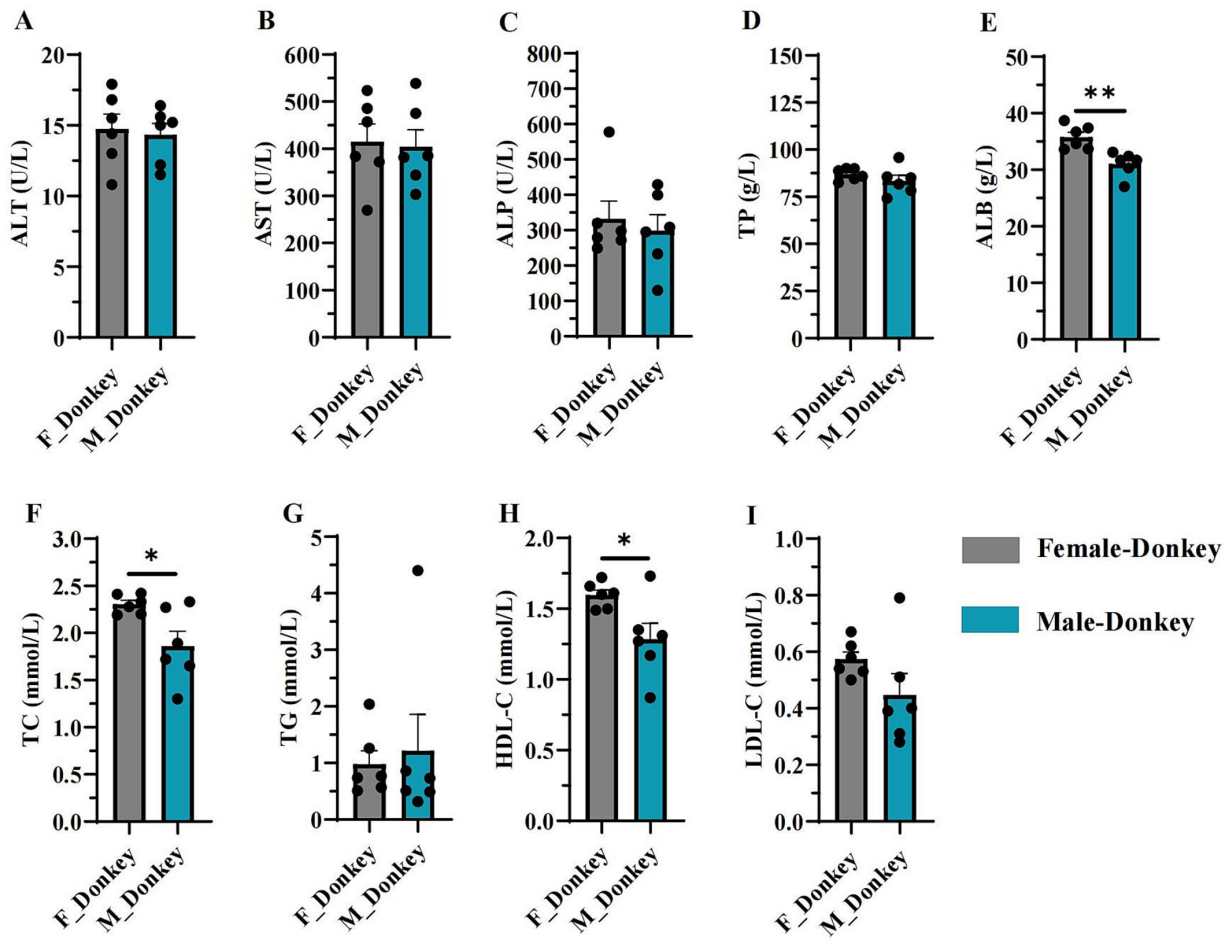
Serum biochemical indices in female and male donkeys. **(A)** ALT level; **(B)** AST level; **(C)** Alkaline phosphatase level; **(D)** *Treponema pallidum* level; **(E)** Albumin level; **(F)** Total cholesterol level; **(G)** Triglyceride level; **(H)** HDL-C level; **(I)** LDL-C level. *p < 0.05, **p < 0.01.

As shown in Figure 1, serum biochemical indices of female and male donkeys were estimated using ALT, AST, ALP, TP, ALB, TC, TG, HDL-C, and LDL-C levels. The levels of ALB (Figure 1E), TC (Figure 1F), and HDL-C (Figure 1H) in the female donkey group were significantly higher (*p* < 0.05) than those in the male donkey group. No significant differences (*p* > 0.05) were observed between male and female groups for the other indices (ALT, AST, ALP, TP, TG, and LDL-C).

### 3.2 Analysis of alpha diversity of duodenal, jejunal, and ileal microbiota in adult female and male donkeys

**Figure 2:**
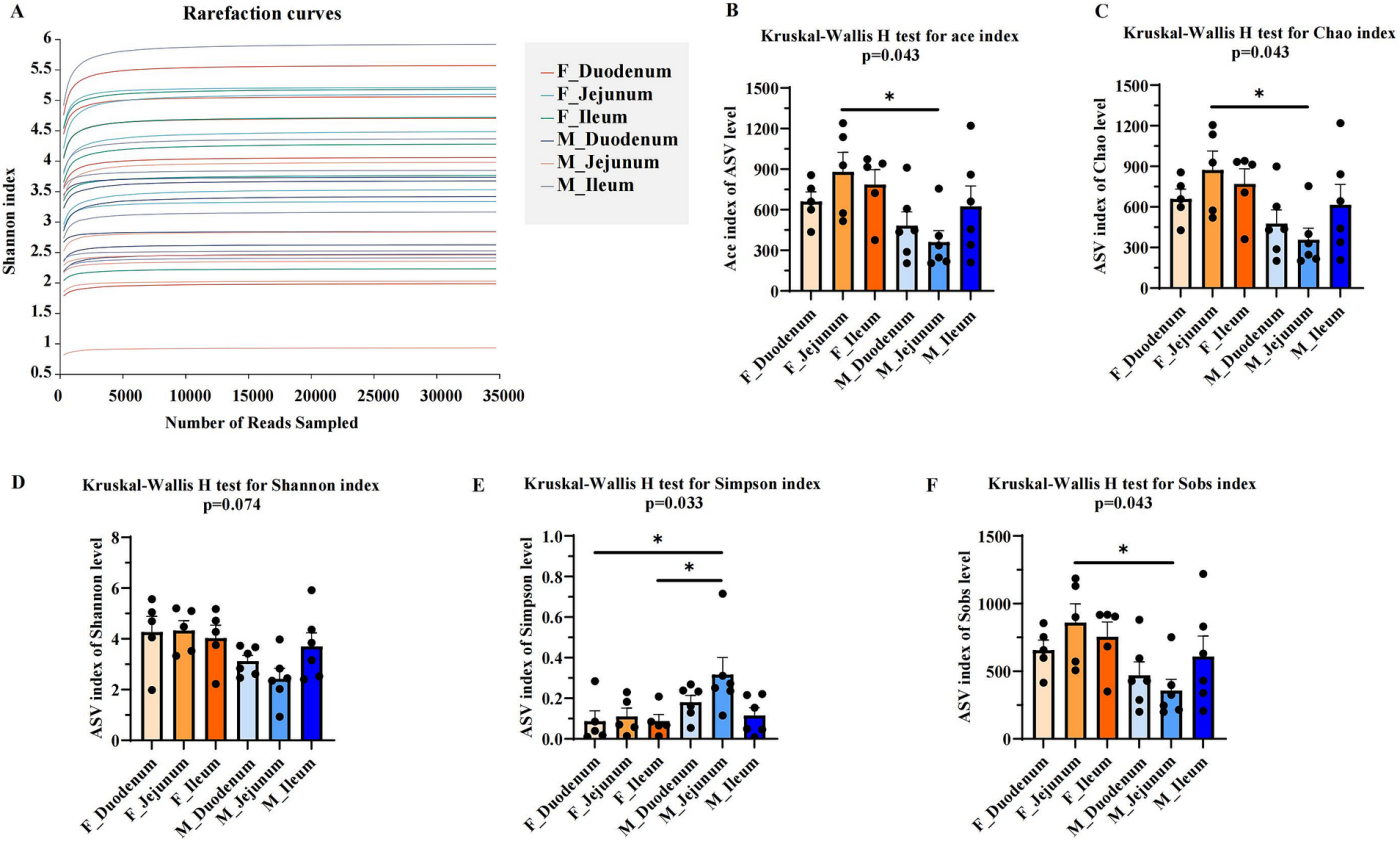
Comparison of microbial diversity indices between two groups. Alpha diversity analyses comparing the microbiota of the two groups based on various microbial diversity indices. **(A)** Rarefaction curves; **(B)** Kruskal-Wallis H test for the Ace index; **(C)** Kruskal-Wallis H test for the Chao index; **(D)** Kruskal-Wallis H test for the Shannon index; **(E)** Kruskal-Wallis H test for the Simpson index; **(F)** Kruskal-Wallis H test for the Sobs index. *p < 0.05.

The alpha diversity of gut microbiota in male and female donkeys was assessed using the Ace, Chao, Shannon, Simpson, and Sobs indices. As shown in Figure 2A, the rarefaction curve levels off as the number of sequences increases, indicating that nearly all bacterial species present were captured across samples. The sequences obtained were then analyzed for diversity. Results showed that the Ace (Figure 2B), Chao (Figure 2C), and Sobs (Figure 2F) indices in the M-Jejunum group were significantly lower (*p* < 0.05) than in the F-Jejunum group, while there were no significant differences in Shannon indices (Figure 2D) between groups. This indicates that there is a higher microorganisms community richness in the jejunum of the female donkey. Additionally, the Simpson index in the F-Duodenum and F-Ileum groups was significantly lower (*p* < 0.05) than in the M-Jejunum group (Figure 2E). This shows that there is a higher microorganisms community diversity of male donkey.

### 3.3 Analysis of gut microbial community of duodenal, jejunal, and ileal microbiota in adult female and male donkeys

**Figure 3:**
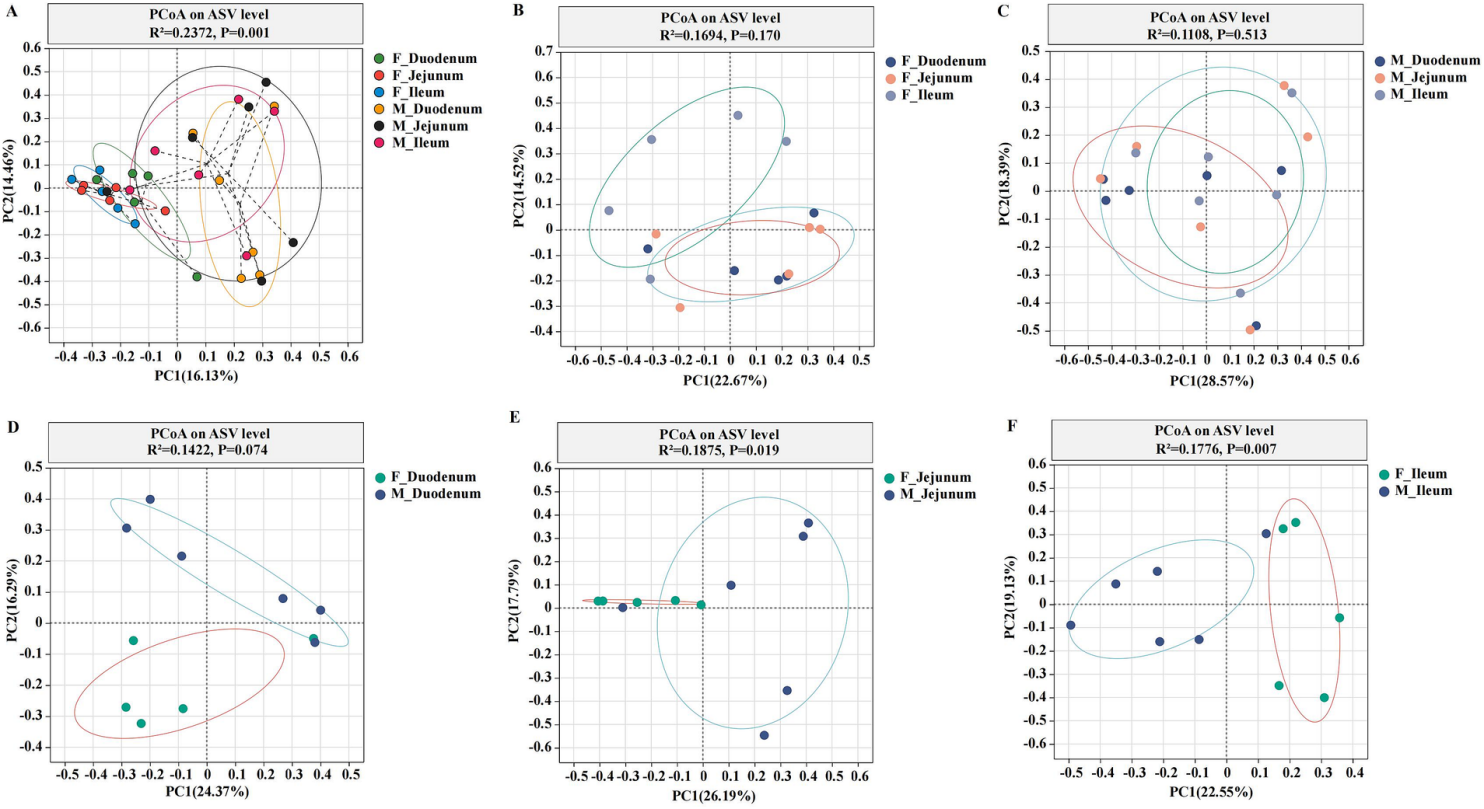
Principal Coordinate Analysis (PCoA) plots showing the gut microbial community structure. **(A)** PCoA of six gut microorganisms; **(B)** PCoA of gut microorganisms in female donkeys; **(C)** PCoA of gut microorganisms in male donkeys; **(D)** PCoA of the duodenum in female and male donkeys (F-Duodenum and M-Duodenum); **(E)** PCoA of the jejunum in female and male donkeys (F-Jejunum and M-Jejunum); **(F)** PCoA of the ileum in female and male donkeys (F-Ileum and M-Ileum). PCoA was performed at the ASV level, with each point representing a sample, and the two clusters indicating a significant difference in community structure between the two groups.

**Figure 4:**
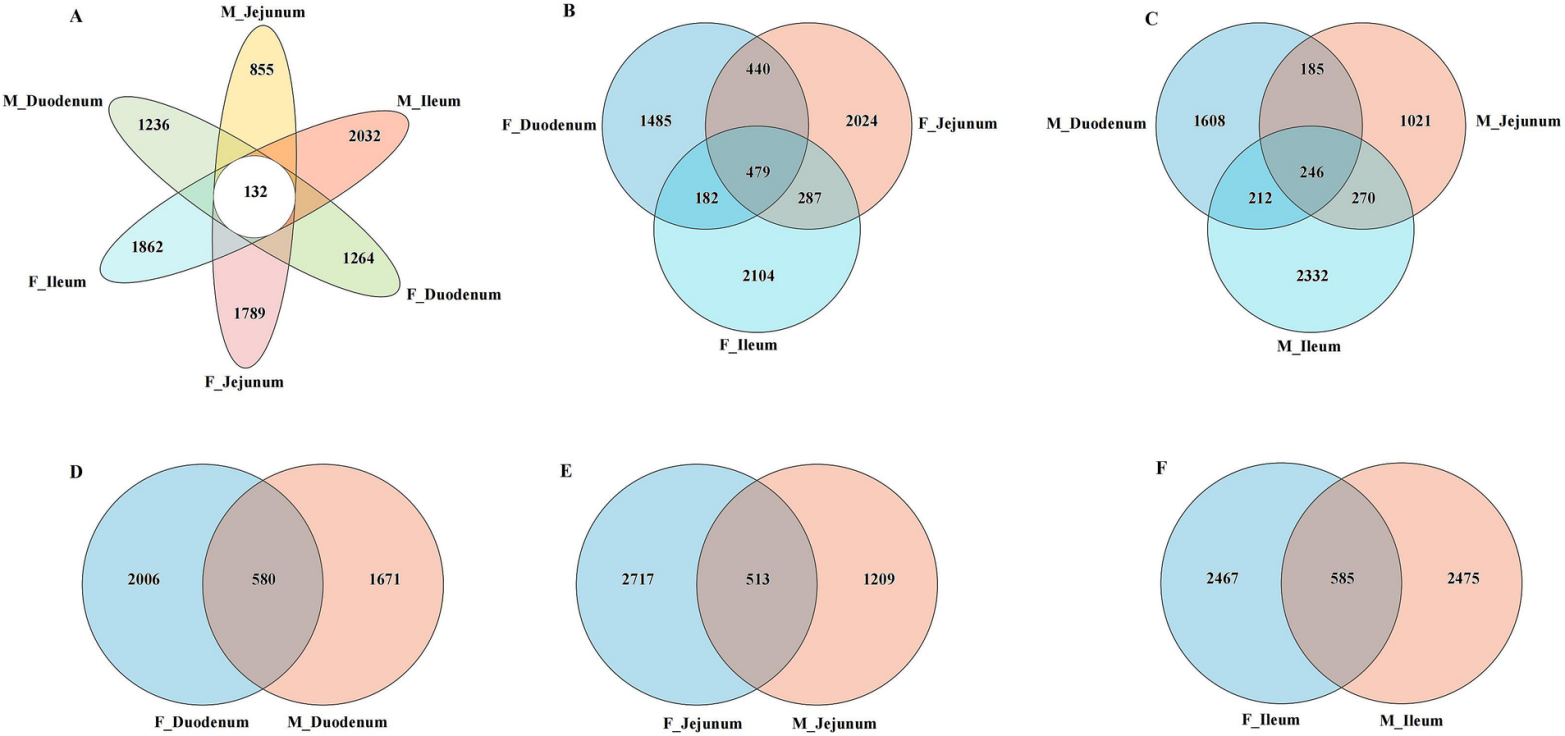
Venn diagrams showing the distribution of gut microbiota ASVs between female and male donkeys. **(A)** Venn analysis of ASVs from six gut microorganisms; **(B)** Venn analysis of ASVs from female donkeys; **(C)** Venn analysis of ASVs from male donkeys; **(D)** Venn analysis of ASVs from the female and male duodenum (F-Duodenum and M-Duodenum); **(E)** Venn analysis of ASVs from the female and male jejunum (F-Jejunum and M-Jejunum); **(F)** Venn analysis of ASVs from the female and male ileum (F-Ileum and M-Ileum).

Principal Coordinate Analysis (PCoA) of *β*-diversity, assessed by Bray–Curtis dissimilarity, confirmed spatial differences in microbial function across different gut locations. As shown in Figure 3A, the gut microbiota in the F-Duodenum, F-Jejunum, F-Ileum, M-Duodenum, M-Jejunum, and M-Ileum groups were clustered separately (R^2^ = 0.2372, *p* = 0.001). The PCoA results showed no significant differences in the microbial structure of the small intestine between adult male (Figure 3B; R^2^ = 0.1694, *p* = 0.170) and female donkeys (Figure 3C; R^2^ = 0.1108, *p* = 0.513); however, there was a potential differentiation in the microbial structure of the duodenum between sexes (Figure 3D; R^2^ = 0.1422, *p* = 0.074), while significant differences were observed in the microbial composition of the jejunum (Figure 3E; R^2^ = 1875, *p* = 0.019) and ileum (Figure 3F; R^2^ = 0.1776, *p* = 0.007) between male and female donkeys. To illustrate the distribution of common and unique ASVs across samples, we used Venn diagrams to represent the bacterial community ASVs. Clustering the valid labels from all samples, the Venn diagrams showed that the six groups shared a community containing 132 ASVs (Figure 4A), while unique ASVs were identified in M-Duodenum (1236), F-Duodenum (1264), M-Jejunum (855), F-Jejunum (1789), M-Ileum (2032), and F-Ileum (1862). Based on microbial analysis of the duodenum, jejunum, and ileum, the bacterial community shared among female donkeys contained 479 ASVs, whereas the male group shared 246 ASVs (Figures 4B,C). Comparing gut segments between adult male and female donkeys, the F-Duodenum and M-Duodenum groups shared 580 ASVs; the F-Jejunum and M-Jejunum groups shared 513 ASVs; and the F-Ileum and M-Ileum groups shared 585 ASVs (Figures 4D–F).

**Figure 5:**
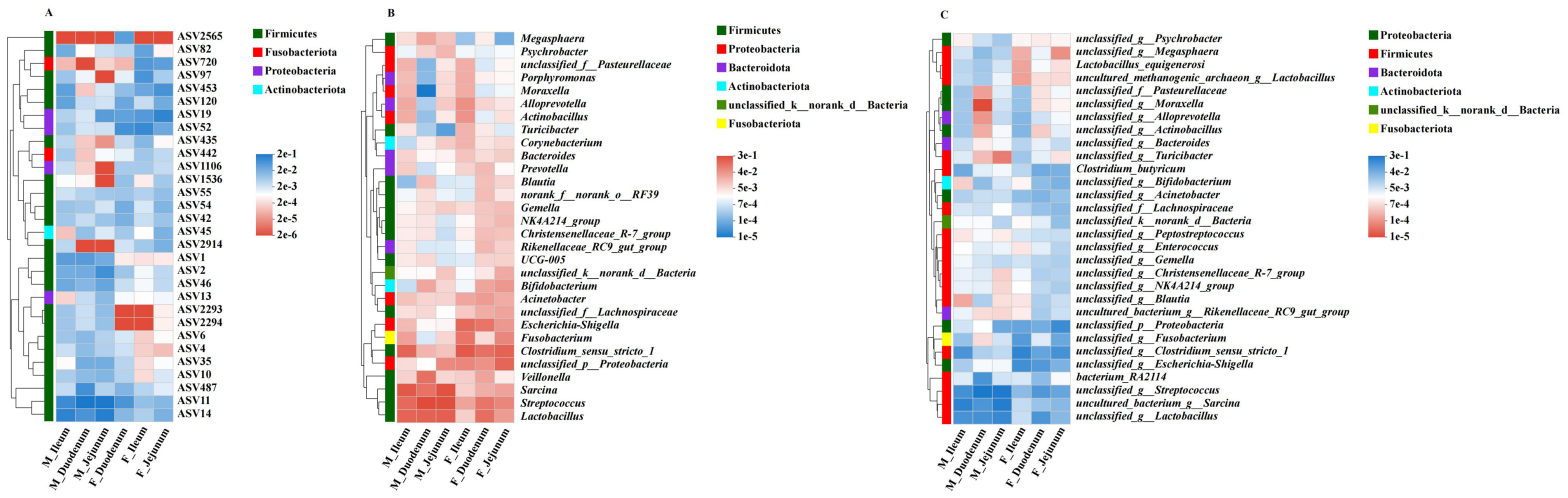
The distribution of important bacterial functions in different groups. **(A)** Correlation of the dominant affected ASVs in the digesta-associated microbiota across different intestinal locations; **(B)** Correlation of different intestinal locations with associated microbiota at the genus level; **(C)** Correlation of different intestinal locations with associated microbiota genera across six phyla.

Based on ASV, phylum, and genus levels, we used heatmaps to illustrate the core species composition across different treatment groups. At the ASV level (Figure 5A), the top 30 species with the highest abundance in the gut-associated microbiota (ASV2565, ASV82, ASV720, ASV97, ASV453, ASV120, ASV19, ASV52, ASV435, ASV442, ASV1106, ASV1536, ASV55, ASV54, ASV42, ASV45, ASV2914, ASV1, ASV2, ASV46, ASV13, ASV2293, ASV2294, ASV6, ASV4, ASV35, ASV10, ASV487, ASV11, and ASV14) were identified among these treatment groups. At the phylum level (Figure 5B), Firmicutes, Proteobacteria, Bacteroidota, Actinobacteriota, unclassified_k_norank_d_Bacteria, and Fusobacteriota dominated the microbial community in all donkeys. At the genus level (Figure 5C), *unclassified_g__Lactobacillus*, *uncultured_bacterium_g__Sarcina*, *bacterium_RA2114*, *unclassified_g__Streptococcus*, and *unclassified_g_Moraxella* were enriched across these treatment groups.

### 3.4 Analysis of differential microbiota in duodenal, jejunal, and ileal microbiota in adult female and male donkeys

**Figure 6:**
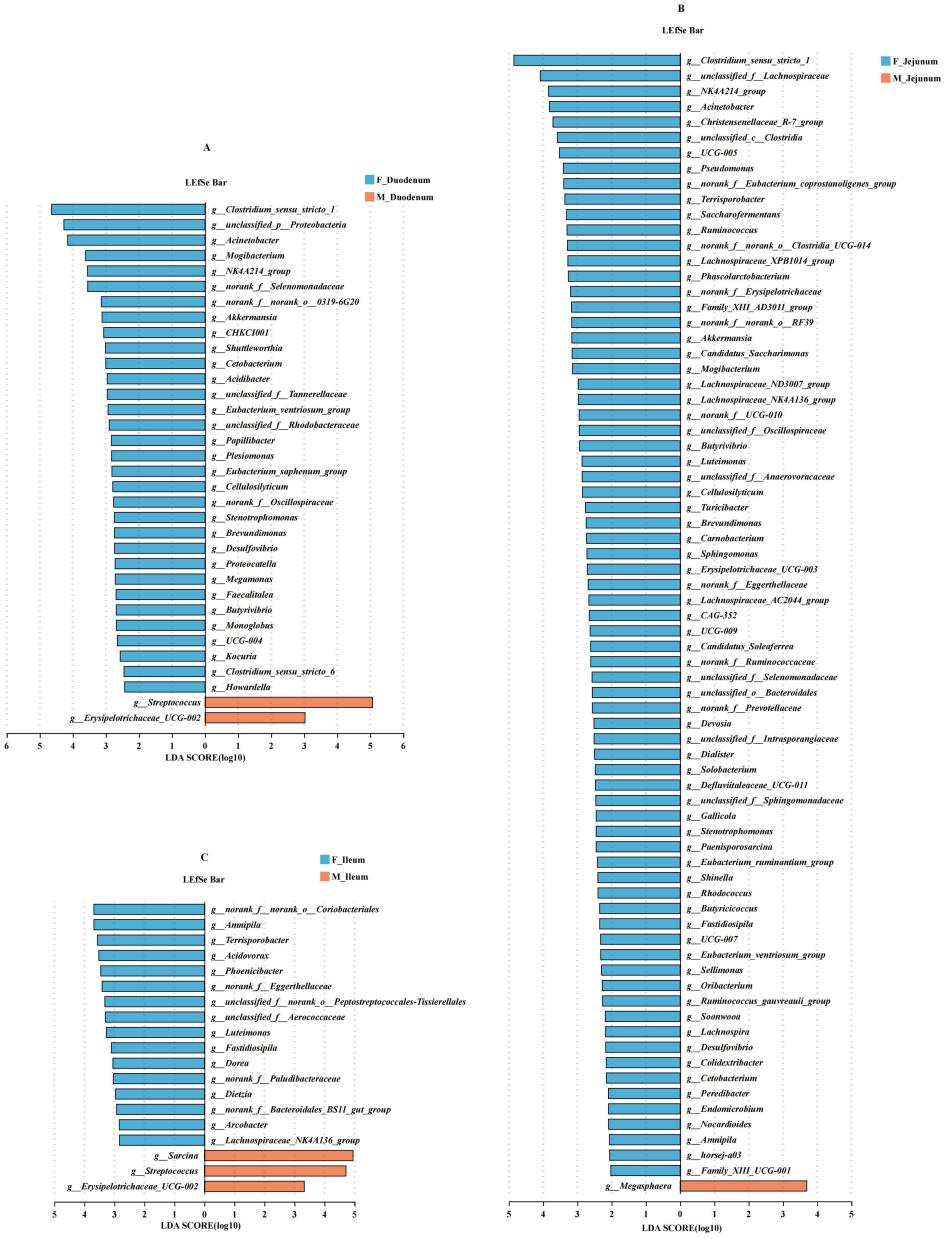
Differentially abundant genera in the gut microbiota of male and female donkeys. **(A)** LEfSe analysis of gut microbiota in the F-Duodenum and M-Duodenum; **(B)** LEfSe analysis of gut microbiota in the F-Jejunum and M-Jejunum; **(C)** LEfSe analysis of gut microbiota in the F-Ileum and M-Ileum.

Next, LEfSe was used to identify bacterial groups that were significantly different between the two management models. As shown in Figure 6A, *Clostridium_sensu_stricto_1*, *Acinetobacter*, *Mogibacterium*, *NK4A214_group*, *Akkermansia*, *Shuttleworthia*, *Cetobacterium*, *Acidibacter*, *Butyrivibrio*, *Monoglobus*, and *Clostridium_sensu_stricto_6* were significantly enriched in the F_Duodenum group, while *Streptococcus* and *Erysipelotrichaceae_UCG-002* were significantly enriched in the M_Duodenum group. In Figure 6B, *Clostridium_sensu_stricto_1*, *NK4A214_group*, *Acinetobacter*, *Christensenellaceae_R-7_group*, *Terrisporobacter*, *Ruminococcus*, *Lachnospiraceae_XPB1014_group*, *Akkermansia*, *Mogibacterium*, *Lachnospiraceae_ND3007_group*, *Butyrivibrio*, *Turicibacter*, *Lachnospiraceae_AC2044_group* and *Lachnospira* were significantly enriched in the F_Jejunum group, while *Megasphaera* was significantly enriched in the M_Jejunum group. In Figure 6C, *Amnipila*, *Terrisporobacter*, *Acidovorax*, *Phoenicibacter*, *Luteimonas*, *Fastidiosipila*, *Dorea*, *Arcobacter*, and *Lachnospiraceae_NK4A136_group* were significantly enriched in the F_Ileum group, whereas *Sarcina*, *Streptococcus*, and *Erysipelotrichaceae_UCG-002* were significantly enriched in the M_Ileum group.

**Figure 7:**
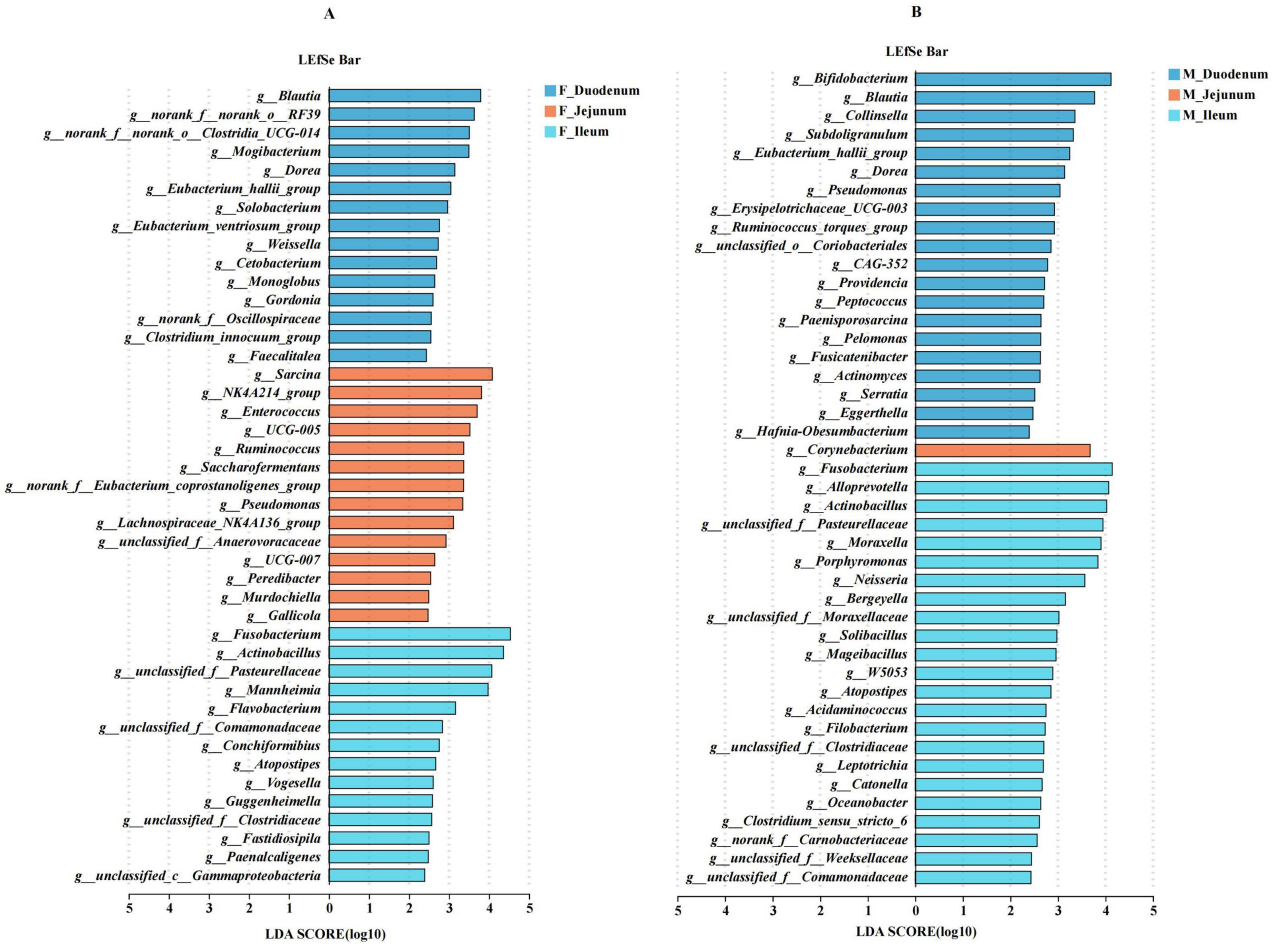
Differentially abundant genera in the gut microbiota of male and female donkeys across the duodenum, jejunum, and ileum. **(A)** LEfSe analysis of gut microbiota in female donkeys across the duodenum, jejunum, and ileum; **(B)** LEfSe analysis of gut microbiota in male donkeys across the duodenum, jejunum, and ileum.

Next, we analyzed differentially abundant microbes across the duodenum, jejunum, and ileum within the same sex. As shown in Figure 7A, *Blautia*, *Mogibacterium*, *Dorea*, *Eubacterium_hallii_group*, *Solobacterium*, *Weissella*, and *Cetobacterium* were significantly enriched in the F_Duodenum group; *Sarcina*, *NK4A214_group*, *Enterococcus*, *UCG-005*, *Ruminococcus*, *Saccharofermentans*, *Pseudomonas*, *Lachnospiraceae_NK4A136_group*, and *Peredibacter* were significantly enriched in the F_Jejunum group; and *Fusobacterium*, *Mannheimia*, *Flavobacterium*, *Conchiformibius*, *Atopostipes*, *Vogesella*, *Guggenheimella*, *Fastidiosipila*, and *Paenalcaligenes* were enriched in the F_Ileum group. As shown in Figure 7B, *Bifidobacterium*, *Blautia*, *Collinsella*, *Subdoligranulum*, *Eubacterium_hallii_group*, *Dorea*, *Pseudomonas*, *Erysipelotrichaceae_UCG-003*, *Ruminococcus_torques_group*, *Providencia*, *Peptococcus*, *Paenisporosarcina*, and *Pelomonas* were significantly enriched in the M_Duodenum group; *Corynebacterium* was significantly enriched in the M_Jejunum group; and *Fusobacterium*, *Alloprevotella*, *Actinobacillus*, *Moraxella*, *Porphyromonas*, *Bergeyella*, *Mageibacillus*, *Atopostipes*, *Filobacterium*, and *Clostridium_sensu_stricto_6* were enriched in the M_Ileum group.

## 4 Discussion

The donkey gut is essential for digestion, with large and complex gut microbes playing a crucial role in breaking down various indigestible polysaccharides (Ma et al., 2022; Zhang et al., 2024; Glatter et al., 2019; Bäckhed et al., 2005). A previous study has shown that herbivores, especially ruminants, host a diverse and specialized microbiota (Bayané and Guiot, 2011). The mammalian gut microbiota is dynamic and complex, plays a fundamental role in responding and adapting to the host environment, which supports host health and normal reproduction (Ley et al., 2008; Li et al., 2022), and supporting immune function and health maintenance (Bertino et al., 2022; Zhao et al., 2013; Maslowski and Mackay, 2011). The normal gut microbiota contributes to host nutrient metabolism, drug and xenobiotic processing, intestinal barrier integrity, immune modulation, and pathogen resistance (Li et al., 2022; Jandhyala et al., 2015). Our findings characterize the gut microbiota of healthy donkeys and reveal differences between male and female donkeys, providing novel insights into donkey gut microbiota.

The mammalian gut microbiota plays a vital role in host metabolism and adaptation (Li et al., 2018). In this study, we examined biochemical indicators in male and female donkey groups and found that ALB, TC, and HDL-C levels were significantly higher in the female group. ALB, a key protein in blood, contributes to vascular endothelial stability, acid–base balance, and the repair of inflammatory damage (Bihari et al., 2020). Additionally, TC transformation is strongly associated with different microbiomes (Bubeck et al., 2023). In previous research on the toxicokinetics of toxins in Dezhou donkeys, including the effects of ochratoxin (OTA) on male donkeys and zearalenone (ZEN) on female donkeys, both of which demonstrated a high absorption rate and slow elimination in Dezhou donkeys (Kang et al., 2023; Qu et al., 2024). Previous research has shown that host genetic influence patterns differ between adult males and females, indicating that gender impacts host metabolism and gut microbiota composition (Zhao et al., 2013; Mueller et al., 2006), which aligns with the findings of our study.

Animal gut microbiota is influenced by both the living environment and sex (de Jonge et al., 2022). Variations in diversity reflect the stability and spatial distribution of the gut microbial environment. In the *α*-diversity results (Ace, Chao, Simpson, Shannon, and Sobs indices), the Ace, Chao, and Sobs indices were significantly lower in the M-Jejunum group than in the F-Jejunum group, indicating differences in jejunal microbial communities between male and female donkeys. Differences in gut microbial composition and the host immune system by sex have been reported (Fushuku and Fukuda, 2008), which aligns with our findings. A previous study showed that PCoA at the ASV level revealed distinct spatial variability of gut microbiota across different gut compartments (Donaldson et al., 2016). In our study, the microbial communities within each intestinal location clustered significantly within both male and female groups, with significant differences observed between male and female donkeys in the jejunum and ileum groups, while microbial composition was more consistent between sexes in the duodenum. Previous research also found that microbial richness and diversity are generally higher in the hindgut than in the foregut (Liu et al., 2019), there were significant differences in terms of dominant bacteria among cecum, ventral colon, and dorsal colon, especially between the cecum and dorsal colon sites (Li et al., 2022), suggesting that potential shifts in bacterial communities warrant further investigation. At the ASVs level, our results indicate that the duodenum, jejunum, and ileum have a high number of unique ASVs, reflecting a rich diversity of microbial species. Similarly, previous research on horses revealed that foregut microbiota varies significantly between sections and individuals (Su et al., 2020). Furthermore, Firmicutes emerged as the most prevalent phylum across all intestinal regions, with its high abundance linked to fiber degradation in herbivores (Liu et al., 2019). In the male donkey group, a unique ASV for Fusobacteriota was identified in the duodenum and one for Proteobacteria in the jejunum. Prior findings suggest that gut microbiota composition varies between sexes, influencing immune properties (Fushuku and Fukuda, 2008). At the phylum level, Firmicutes and Proteobacteria dominated the microbial community in all donkeys, with other identified phyla including Bacteroidota, Actinobacteriota, unclassified_k_norank_d_Bacteria, and Fusobacteriota. Most Firmicutes produce butyric acid that is not absorbed by the intestine, but it provides energy and promotes the development of intestinal epithelial cells (Guo et al., 2023). Interestingly, Steelman et al. (2012) found that bacterial communities in equine gut samples were dominated by Firmicutes and Verrucomicrobia, followed by Bacteroidetes, Proteobacteria, and Spirochaetes, which aligns with our findings. In male donkeys, *Sarcina*, *Streptococcus*, and *Lactobacillus* showed higher relative abundance; these genera within Firmicutes are known to influence gut health differently between sexes. Both *Lactobacillus* and *Streptococcus* have evidence of potential beneficial functions in relation to lipid metabolism (Liu et al., 2019; Ma et al., 2022), illustrating that they play an important role in maintaining intestinal health. Firmicutes is also known to promote short-chain fatty acid (SCFA) production, which supports fat accumulation (Ley et al., 2005). The presence of *Sarcina* often correlates with health complications (Makovska et al., 2023), however, a previous study showed the increased relative abundance of *Sarcina* significantly affected the immune response in rats (Vega-Magaña et al., 2020), our study shows that it seems to appear to be a common member of the gut microbiota. *Streptococcus* has been linked to infectious diseases, triggered by arginine and inhibited by carbon metabolites (Gruening et al., 2006). In contrast, *Lactobacillus* includes acid-tolerant mutualistic bacteria that selectively inhibit pathogens and produce free bile acids to support fat metabolism (Corzo and Gilliland, 1999). The interaction between *Sarcina* and *Lactobacillus* may enhance metabolism in male donkey intestines.

At the genus level, our study found that *unclassified_g_Moraxella* was enriched in the M-Duodenum group. Previous research has shown that stomach bacterial communities are often dominated by Firmicutes, Proteobacteria, and Bacteroidetes, with *Lactobacillus* spp., *Streptococcus* spp., and *Moraxella* spp. as prominent genera (Perkins et al., 2012). These findings indicate differences in microbiota between male and female donkey groups, particularly within Firmicutes. Additionally, *Bifidobacterium* was a dominant genus in the M-Duodenum group, and *Clostridium*, *Lactobacillus* and *Enterococcus* have been noted as primary mucosa-associated genera in the small intestine (Swidsinski et al., 2005). Clostridium species in the gut play a vital role in the production of vitamins and short-chain fatty acids, the maintenance of gut homeostasis and the shaping of the mucosal immune system (Marathe et al., 2014). Therefore, there is evidence to speculate that male donkeys have better digestive capacity due to these microbial floras. LEfSe analysis revealed *g_Fusobacterium* as the dominant genus in the F-Ileum group, consistent with findings from fecal studies in diverse horse groups where *Fusobacterium* spp. was prevalent (Costa et al., 2012). This led us to hypothesize that microbial specificity in donkeys may closely relate to gut location and gender. In the M-Ileum group, *g_Sarcina* and *g_Streptococcus* were predominant, with Sarcina spp. known as opportunistic pathogens found in diverse mammalian hosts (Makovska et al., 2023). Notably, *g_Streptococcus* was more abundant in the M-Duodenum than in the F-Duodenum group, differing from prior studies where Lactobacillus was dominant in the foregut and Streptococcus in the hindgut (Liu et al., 2019). In addition, *g_Clostridium_sensu_stricto_1* dominated in the F-Jejunum group, while *g_Megasphaera* was predominant in the M-Jejunum group. Clostridium represents a highly diverse genus with both beneficial and pathogenic species (DuPont and DuPont, 2011). A study shows that a potentially pivotal lipid-lowering role of *Megasphaera* in the gut microbiota (Gao et al., 2022). Further research is needed to explore the link between regional colonization differences in the Dezhou donkey gut and metabolic functions, informing future feeding management and promoting intestinal health in donkeys.

## 5 Conclusion

This study provides novel insights into the spatial distribution of foregut microbial communities in healthy male and female Dezhou donkeys. Gender was identified as a significant factor influencing gut microbial composition, with notable differences in predicted microbiota functions. Additionally, the microbial composition exhibited distinct distribution patterns across the duodenum, jejunum, and ileum within each gender. These findings enhance our understanding of donkey gut microbiology and clarify the role of the gut in maintaining overall health.

## Data Availability

The raw sequencing data is publicly available. This data can be found here: https://www.ncbi.nlm.nih.gov/, accession number: PRJNA1257860.

## Footnotes

^1^ http://www.r-project.org/
